# Prediction of Rock Unloading Strength Based on PSO-XGBoost Hybrid Models

**DOI:** 10.3390/ma17174214

**Published:** 2024-08-26

**Authors:** Baohua Liu, Hang Lin, Yifan Chen, Chaoyi Yang

**Affiliations:** 1 School of Resources and Safety Engineering, Central South University, Changsha 410083, China; 215501002@csu.edu.cn (B.L.); yangchaoyiyn@126.com (C.Y.); 2 Yunan Diqing Non-ferrous Metals Co., Ltd., Shangri-La 674400, China

**Keywords:** rock strength, unloading, decreasing confining stress and increasing axial stress, prediction modeling, machine learning

## Abstract

Rock excavation is essentially an unloading behavior, and its mechanical properties are significantly different from those under loading conditions. In response to the current deficiencies in the peak strength prediction of rocks under unloading conditions, this study proposes a hybrid learning model for the intelligent prediction of the unloading strength of rocks using simple parameters in rock unloading tests. The XGBoost technique was used to construct a model, and the PSO-XGBoost hybrid model was developed by employing particle swarm optimization (PSO) to refine the XGBoost parameters for better prediction. In order to verify the validity and accuracy of the proposed hybrid model, 134 rock sample sets containing various common rock types in rock excavation were collected from international and Chinese publications for the purpose of modeling, and the rock unloading strength prediction results were compared with those obtained by the Random Forest (RF) model, the Support Vector Machine (SVM) model, the XGBoost (XGBoost) model, and the Grid Search Method-based XGBoost (GS-XGBoost) model. Meanwhile, five statistical indicators, including the coefficient of determination (R^2^), mean absolute error (MAE), mean absolute percentage error (MAPE), mean square error (MSE), and root mean square error (RMSE), were calculated to check the acceptability of these models from a quantitative perspective. A review of the comparison results revealed that the proposed PSO-XGBoost hybrid model provides a better performance than the others in predicting rock unloading strength. Finally, the importance of the effect of each input feature on the generalization performance of the hybrid model was assessed. The insights garnered from this research offer a substantial reference for tunnel excavation design and other representative projects.

## 1. Introduction

The earliest research on rock mechanics focused on the mechanical response of rocks under loading conditions, with the goal of reflecting on the failure mechanism of rocks caused by loading, and significant research progress has been made in this field at the theoretical, experimental, and numerical levels [1,2,3,4]. However, with the proposal of the national strategy of “asking for space and resources underground”, the number of underground space-related projects is daily increasing, and unloading stress environments are frequently encountered during rock engineering construction. For example, numerous engineering practices have shown that rocks and rock masses are usually in an unloaded stress environment when excavating tunnels [5], in which the stress field of the surrounding rock vary and are accompanied by the rock deformation, resulting in various degrees of degradation of the mechanical parameters. This further aggravates the rock deformation, which leads to the mechanical response under unloading conditions to deviate from the results of the existing theory of rock failure under loading conditions. As a consequence, the focus of rock mechanics research has gradually come to include unloaded rock mechanics.

To investigate the mechanical properties of rock under unloading conditions, scholars and experts at home and abroad have carried out a considerable amount of research in recent years. First of all, on the basis of years of practical engineering experience and related research, Ha [6] believes that rocks are more sensitive under unloading than loading conditions, and they suggested that unloaded rock mechanics should be studied from a practical view. Lau and Chandler [7] also emphasized that the mechanical parameters derived from the loading conditions did not correspond to the actual excavation of the rock mass, whereas those obtained through the unloading path were more accurate. Subsequently, the mechanical behavior of rocks under unloading conditions has been intensively studied. For example, Swanson [8] and Crouch [9] carried out an exploratory study on unloaded rock mechanics through an experimental method. In the same way, Zivaljevic and Tomanovic [10] analyzed the deformational behavior of soft rocks after unloading. Abi et al. [11] investigated the warning signs of rock failure under unloading conditions based on the evolution law of rock entropy. Gu et al. [12] proposed a new mechanical model for dynamic unloading under hydrostatic pressure. Manouchehrian and Cai [13] simulated the unstable rock failures under poly-axial unloading conditions by a numerical method. He and Zhao [14] showed that granites show varying degrees of damage as the unloading rate increases. Chen et al. [15] observed that crack volume growth strain and crack growth rate increased with unloading rate. Li et al. [16] modeled brittle rock unloading and found that low unloading rates weakened the kinetic effect and that nonlinear unloading paths outperformed linear paths in releasing kinetic energy. Bing et al. [17] pointed out that the destructive strain of granite during unloading was less than that during loading, and the difference was increasing with the increase in confining stress.

In addition, the factors affecting the mechanical properties of rocks under unloading conditions have likewise received extensive attention. Fotovvat et al. [18] compared the influences of the unloading rate and initial stress ratio on the unloading failure by constant deviator stress tests. Huang et al. [19] studied the deformation mechanism of deep soft rock and determined the relationship between the unloading rate and post-peak brittleness. Xu et al. [20] conducted triaxial unloading tests on marble at different unloading rates, revealing the relationship between the unloading strain rate and the form and degree of damage. Cao et al. [21] revealed the quantitative relationship between unloading vibration characteristics, the unloading rate, and other related factors. Li et al. [22] demonstrated the relationship between the unloading rate of confining stress and cracking and stress extension. Wang et al. [23] investigated the effect of the unloading rate on the axial and radial strains of sandstone and found that radial deformation was more sensitive to the unloading process. Zhang et al. [24] indicated the effect of confining the stress difference and unloading rate on the peak stress. Si and Gong [25] declared that the lower the unloading rate, the higher the rock strength and the easier it is to accumulate elastic energy.

From the above, it can be noticed that there are many factors affecting the mechanical properties of rocks under unloading conditions, and most of the conclusions are qualitative analyses of the unloading mechanical behavior of rocks with a single influencing factor, while quantitative analyses of the unloading strength characteristics of rocks with comprehensive considerations of multiple factors have been rarely reported. In this regard, this study firstly constructed a database by collecting the unloading test data of different types of rocks and established a rock unloading strength prediction model based on the XGBoost algorithm, which utilizes the PSO optimization algorithm to complete hyperparameter optimization during the model training process. A comprehensive comparison was organized between the prediction results by this hybrid model and other mainstream models, and multiple evaluation indicators were adopted to assess the feasibility and generalization ability of the hybrid model. Finally, the importance of the input features was analyzed, which provides some reference for the study of the mechanical properties of rock unloading.

## 2. Rock Unloading Path

**Figure 1:**
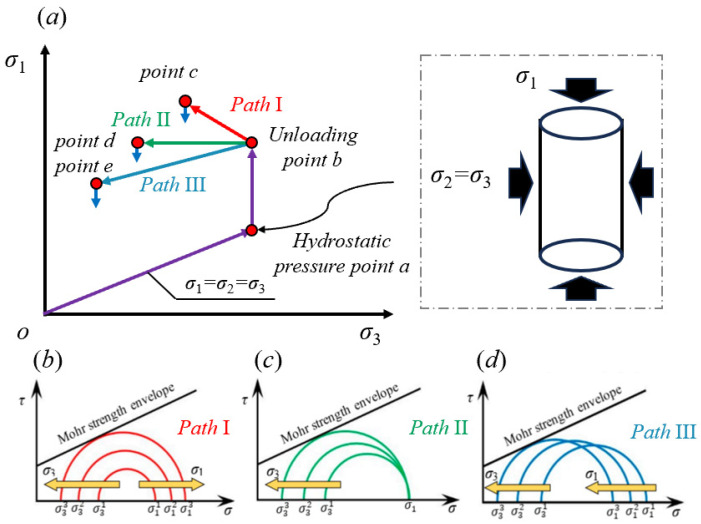
Descriptions of the different unloading paths [27]. (**a**) The commonly used stress unloading paths in the laboratory; (**b**) the unloading failure model for Path I; (**c**) the unloading failure model for Path II; and (**d**) the unloading failure model for Path III.

As mentioned earlier, during the excavation process of tunnels and other projects, the rock mass is in an unloading condition. The mechanical properties of the rock mass under unloading conditions are different from those under conventional loading conditions. Therefore, the conventional triaxial test does not fully describe the unloading mechanical properties of underground rock, and the rock mechanical properties obtained by unloading tests are more in accordance with the actual situation of the tunnel surrounding rock. Depending on the stress state of the surrounding rock during tunnel excavation, the commonly used stress unloading paths in laboratory testing can be categorized into three types: (1) Path I, where the unloading confining stress is subjected to loading axial stress; (2) Path II, where the unloading confining stress is subjected to constant axial stress; and (3) Path III, where the unloading confining stress is subjected to unloading axial stress. Figure 1 shows the manner in which the stresses are unloaded in the experimental implementation, as well as the failure process under different loading paths. These are detailed more specifically below.

(1) Path I: The confining stress decreases while the axial stress keeps increasing (i.e., *o*~*a*~*b*~*c* in Figure 1a). During this loading path, *σ*_1_ = *σ*_2_ = *σ*_3_ is firstly increased from Point *o* to Point *a* (hydrostatic pressure). Then, keeping *σ*_2_ = *σ*_3_ constant and increasing the axial stress *σ*_1_ to the unloading Point *b*, unloading *σ*_2_ = *σ*_3_ is finally achieved while *σ*_1_ continues to increase until rock failure Point *c*.
(2) Path II: The confining stress decreases while the axial stress stays constant (i.e., *o*~*a*~*b*~*d* in Figure 1a). The loading procedure of *o*~*a*~*b* is the same as for Path I. Finally, unloading *σ*_2_ = *σ*_3_ is achieved while *σ*_1_ stays constant until rock failure Point *d*.
(3) Path III: The confining stress decreases while the axial stress also decreases (i.e., *o*~*a*~*b*~*e* in Figure 1a). The loading procedure of *o*~*a*~*b* is the same as for Path I. Finally, both confining stress *σ*_2_ = *σ*_3_ and *σ*_1_ unload until rock failure Point *e*.

In terms of the failure model, the center of the Mohr circle in Path I (see Figure 1b) remains constant, and the diameter increases until it is tangent to the strength envelope. In contrast, the Mohr circle in Path II also increases in diameter, but the center point is shifted to the left until it is tangent to the envelope because the axial stress remains constant (see Figure 1c). As shown in Figure 1d, the Mohr circle in Path III has a constant diameter and moves to the left until rock failure. In summary, the failure model of rocks under unloading conditions greatly depends on the pattern of axial stress variation. Considering the fact that tunnel excavation leads to a decrease in rock confining stress and an increase in axial stress, which is the shortest and most dangerous case of rock failure [26], Path I was chosen as the object and focus of this study.

## 3. Model Construction

### 3.1. XGBoost Model

XGBoost (extreme gradient boosting) is an optimized, distributed gradient boosting decision tree algorithm designed to achieve higher efficiency, performance, and accuracy. Its core principle is based on the gradient boosting (GB) framework, which works by constructing multiple weak predictive models (usually decision trees) and combining them into a stronger predictive model. The expression is

(1) $$y_{i}^{\left(M\right)}=\sum_{j=1}^{M}f_{i}\left(x_{i}\right),$$

where *x_i_* is the *i*-th sample feature value; *y_i_* is the predicted value of the model for the *i*-th sample; *M* is the number of regression trees; and *j* is the *j*-th regression tree.

It improves the execution efficiency while guaranteeing the predictive performance of the model and has significant advantages when dealing with small sample datasets. The detailed principle of XGBoost can be shown as follows:

(1) Gradient boosting framework

The XGBoost model was developed based on the gradient boosting framework. The core idea is to construct a series of weak learners by iterative means, which will, in turn, correct the errors of the previous model. In each iteration, a new model is trained to fit the residual (i.e., the difference between predicted and actual values by the current model). Such a residual can be regarded as the “gradient” of the current model; hence, the term gradient boosting.

(2) Objective function

The objective function of XGBoost consists of two parts: a loss function and a regularization term. The loss function measures the gap between the predicted and actual values of the model, while the regularization term is used to control the complexity of the model and to prevent overfitting. This helps to make the model more generalized and improves the prediction performance.

(3) Tree construction

An additive model is used in the XGBoost model, where a new tree is added in each iteration, and the output of the new tree is added to the output of the previous model. It also performs pruning during tree construction, which means that it will decide whether to continue splitting nodes based on a regularized loss function. This prevents producing overly complex models on small sample datasets.

(4) Approximate histogram algorithm

To improve computational efficiency, the XGBoost model uses an approximate histogram algorithm to find the best split point. Instead of performing an exact traversal of the feature values for each instance, it divides the feature values into intervals to form a histogram, which allows for a quick estimation of the effect of the split point within each interval. Moreover, it allows the use of an adaptive learning rate, which means that, in each iteration, the model can adjust the magnitude of its updates. In small sample data, this mechanism helps to avoid excessive model updates and maintains the stability and accuracy of the model.

(5) Parallel processing

The XGBoost model implements parallel computation in the feature dimension. When calculating the gain of each feature, it can run on different processors at the same time, thus accelerating the training process. Even on small sample datasets, it can utilize efficient algorithm design and parallel computing capabilities to quickly complete the training process, saving time and computational resources.

(6) Sub-sampling and column sampling

To increase the robustness of the model and prevent overfitting, the XGBoost model uses a sub-sampling (row sampling) and column sampling (feature sampling) strategy, which means that only a portion of the data and features are randomly selected for modeling in each iteration. In a small sample dataset, this randomness simulates the effect of a larger dataset and prevents the model from relying too much on certain features or samples.

(7) Missing value processing

The XGBoost model has a built-in missing value processing mechanism, where the algorithm automatically learns the optimal orientation of missing values when they are encountered, thus avoiding the need for complex missing value padding in the preprocessing stage. This is especially important in small sample data as each sample may carry important information and any missing data may lead to a decrease in model performance.

(8) Information on second-order derivatives

In addition to using first-order derivatives (gradients), the XGBoost model uses second-order derivatives (Hessian matrices), which provide information about the curvature of the loss function and help with faster convergence.

With the above mechanism, the XGBoost model was able to provide high performance model training and prediction while maintaining high prediction accuracy.

### 3.2. PSO-XGBoost Hybrid Model

The XGBoost model reduces the risk of overfitting and improves the generalization ability and stability of the model through various mechanisms, which makes it perform well when dealing with small sample datasets. Nonetheless, small sample datasets may still suffer from statistical instability and model bias. For example, different values of hyperparameters may produce different results. Therefore, a combination of other strategies, such as data enhancement, may be required in practical applications. In this case, using the particle swarm optimization (PSO) algorithm to refine the parameters of the XGBoost model is an effective machine learning practice.

First of all, the XGBoost model has several hyperparameters, such as learning rate, tree depth, regularization parameters, and subsample proportion. Together, these parameters form a high-dimensional search space, which may be inefficient and difficult to use in achieving global optimality by manual tuning or when using grid and random searches. The relationships between hyperparameters are also often nonlinear and may even be nonconvex. This means that there may be multiple local optimal solutions in the parameter space, and the PSO algorithm has a better global search capability and is able to find a location closer to the global optimal solution in a nonlinear and nonconvex environment, which can reduce the subjective factors and provide a more objective and systematic parameter selection. In addition, the PSO algorithm is a population-based heuristic search method that mimics the flocking behavior of birds and fishes to optimize the search process through inter-particle interactions and information sharing, balancing the exploration of unknown regions with the exploitation of known good solutions [28]. This balance is crucial for hyperparametric optimization because it can help avoid falling into local optima while speeding up the optimization search process. In addition, the PSO algorithm can be easily applied to problems of different scales, from small datasets to large datasets. Finally, the demonstrable success of PSO in similar publication references made it the preferred choice for our study.

**Figure 2:**
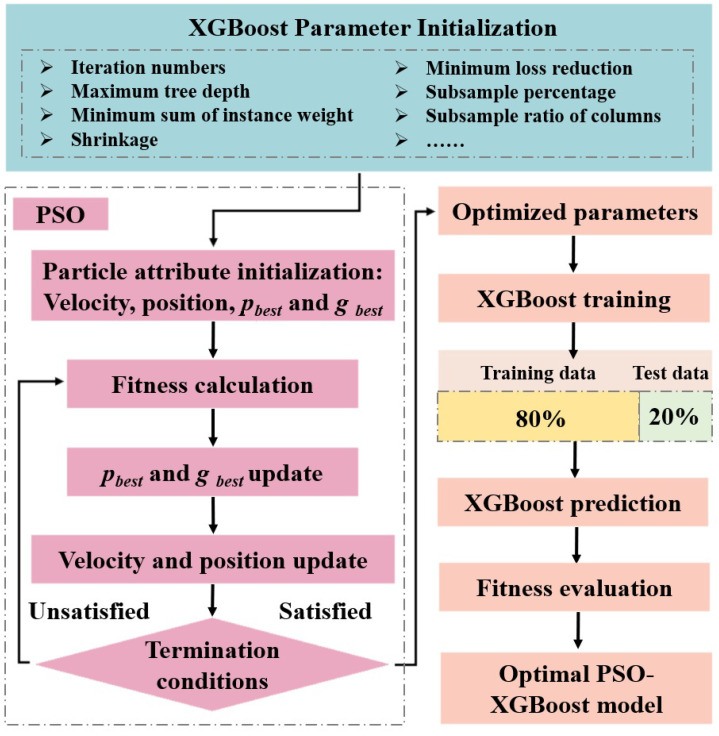
Construction flow of the PSO-optimized XGBoost model.

The main processes of the model construction and optimization are shown in Figure 2, in which the initial XGBoost parameters are presented in the light blue frame, the pink flowchart marks the process of the PSO optimization, and the orange flowchart details the construction of a XGBoost prediction model. In addition, light yellow and light green are also used to differentiate the training set and testing set of the data.

## 4. Model Validation

### 4.1. Database Construction

The dataset used in this study was obtained from the published national and international literature, covering common rock categories of engineering excavations such as sandstone, coal, mudstone, shale, marble, granite, slate, and schist, etc., which were axially loaded while unloading confining stress (Path I in Figure 1). Literature sources, as well as the main parameters affecting the unloading strength of the rock (including lithology, uniaxial compressive strength *UCS*, initial confining stress *σ*_30_, unloading velocity *v*, damage confining stress *σ*_3*p*_, and peak strength *σ*_1*p*_), are presented in Table 1.

Before model validation, the dataset needs to be pre-divided into a training set and a test set, where the training set is used for model training and the test set is used to validate the reliability and evaluate the generalization performance of the trained model. Based on the 134 sets of test data collected, a dataset for rock unloading strength was established, and this study then randomly divided it according to the widely acceptable 8:2 ratio, which ensures that the training set and test set are independent of each other without any intersection. Taking *UCS*, *σ*_30_, *v*, and *σ*_3*p*_ as the input features and *σ*_1*p*_ as the output features, a prediction model of the rock unloading strength based on the hybrid PSO-XGBoost algorithm was therefore achieved, as shown in the flowchart in Figure 2.

### 4.2. Evaluation Indicators

In order to prove that the PSO-XGBoost hybrid model has better prediction and generalization performance, four other commonly used models, i.e., the Random Forest (RF) model, the Support Vector Machine (SVM) model, the XGBoost (XGBoost) model, and the XGBoost hybrid model based on the Grid Search Method (GS-XGBoost) model, were employed in this study to carry out rock unloading strength predictions under the same conditions.

Meanwhile, as shown in Table 2, five statistical indicators containing the coefficient of determination (R^2^), mean square error (MSE), root mean square error (RMSE), mean absolute error (MAE), and mean absolute percentage error (MAPE) were applied to quantify the performance of each model. R^2^ denotes the accuracy of the model in fitting the data, and the closer its value is to 1, the better the model fits the data. MSE, RMSE, MAE, and MAPE reflect the deviation degree of the predicted values from the tested values.

### 4.3. Model Evaluation

**Figure 3:**
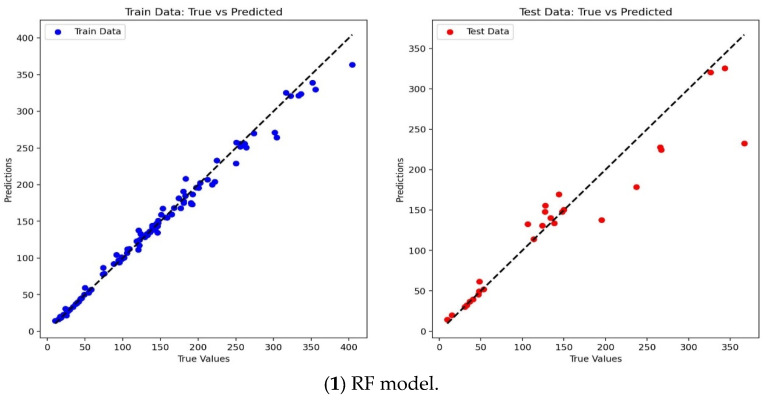

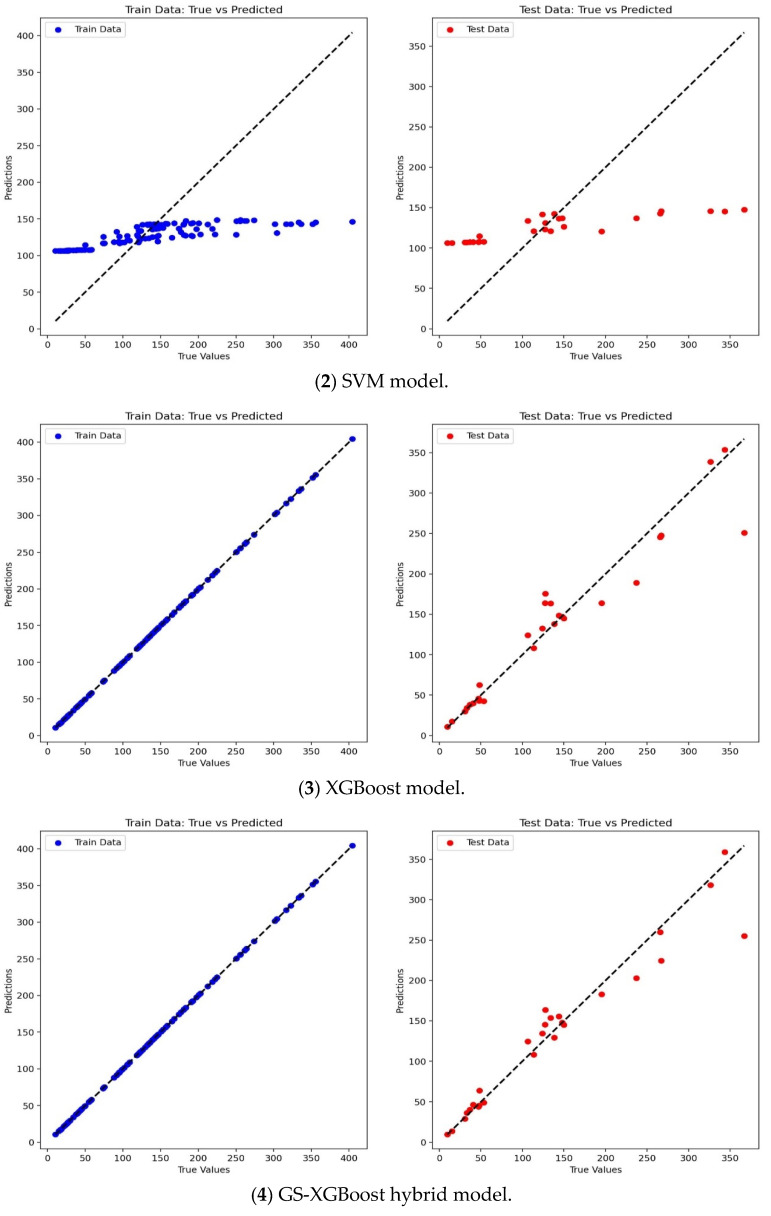

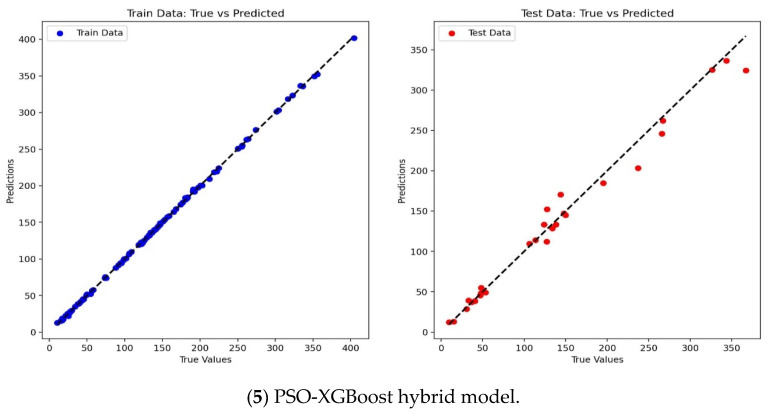
Comparison of the training results and test results of different models.

**Figure 4:**
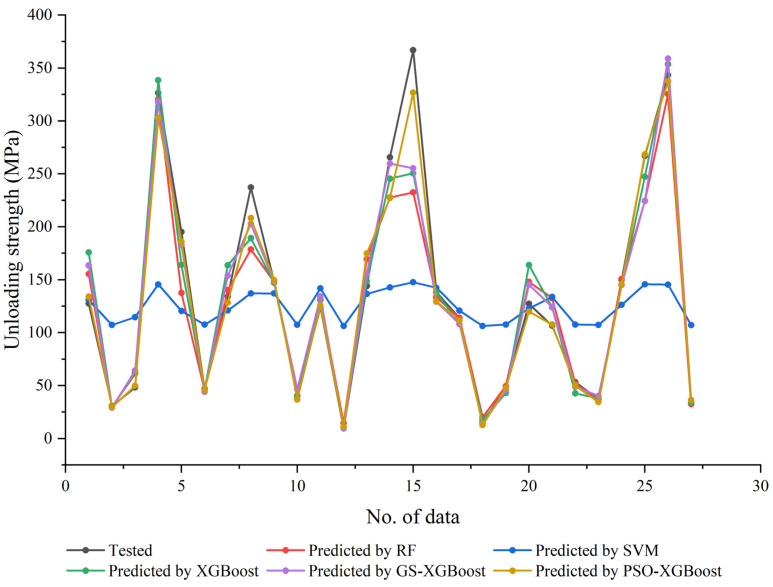
Comparison of the prediction results of different models.

It was clear that, as shown in Figure 3, the XGBoost model, the GS-XGBoost hybrid model, and the PSO-XGBoost hybrid model can achieve satisfactory prediction effectiveness, both for the training set data and the test set data, while the predicted values by the RF model deviated from the experimental values to some extent, and the SVM model performed the worst. When comparing the predicted values of all models in the same plot (Figure 4), it can be more intuitively found that, except for the blue curve represented by the SVM model, all the other four curves showed better agreement with the black curve (tested values). Specifically, the predicted values by the PSO-XGBoost hybrid model were numerically closest to the tested values with the highest degree of consistency, which was followed by the GS-XGBoost hybrid model, the XGBoost model, the RF model, and, finally, the SVM model.

**Figure 5:**
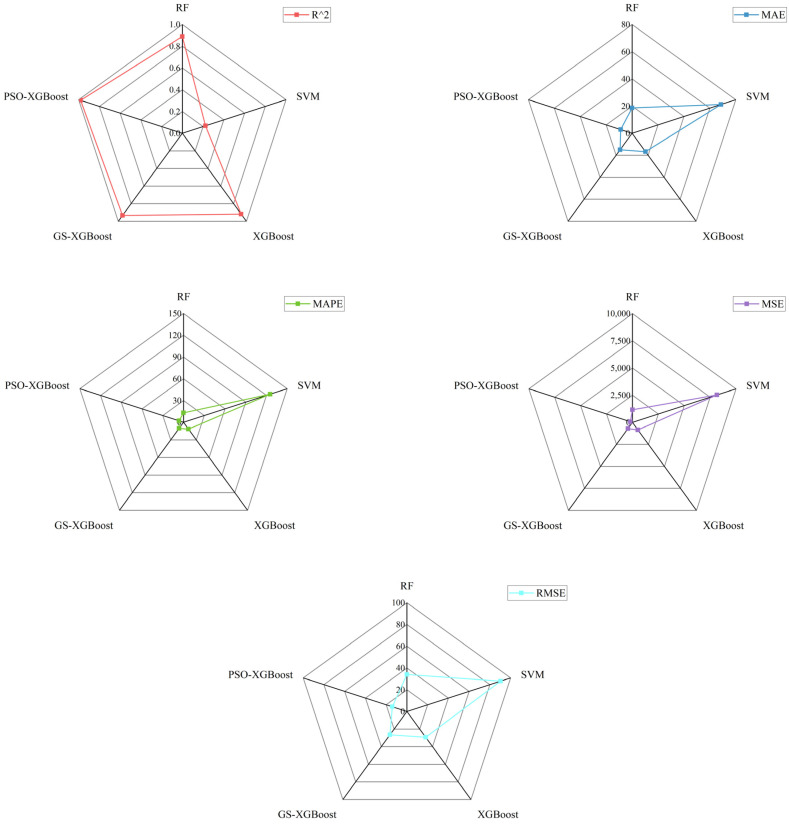
Comparison of the evaluation indicators of different model prediction results.

As shown in Figure 5, the order of R^2^ of the prediction results by the five models was as follows: PSO-XGBoost hybrid model > GS-XGBoost hybrid model > XGBoost model > RF model > SVM model. The order of the remaining evaluation indicators was as follows: PSO-XGBoost hybrid model < GS-XGBoost hybrid model < XGBoost model < RF model < SVM model. Consequently, the predictive performance of the five models was ranked as PSO-XGBoost hybrid model > GS-XGBoost hybrid model > XGBoost model > RF model > SVM model. This is consistent with the conclusions drawn in Figure 3 and Figure 4. In terms of the numerical values of the evaluation indicators (Table 3), the predictive performances of the PSO-XGBoost hybrid model in the training set were better than those of the RF model, the SVM model, the XGBoost model, and the GS-XGBoost hybrid model, with R^2^, MAE, MAPE, MSE, and RMSE values of 0.98, 8.80, 6.51, 198.10, and 14.07, respectively. The value of R^2^ of the PSO-XGBoost hybrid model prediction results was quite close to 1, and the rest of the evaluation indicators were all smaller than the other four models, which indicates the excellence of the PSO-XGBoost hybrid model proposed in this study. This was followed by the GS-XGBoost hybrid model and the XGBoost model, respectively, which do not differ much regarding R^2^ and are both located in the interval [0.9, 0.95], proving their good predictive performance. However, with regard to the values of MAE, MAPE, MSE, and RMSE, the GS-XGBoost hybrid model was smaller than the XGBoost model. The RF model corresponded to an R^2^ of 0.89, which is slightly smaller than 0.9, and the prediction results were general. The SVM model prediction results, on the other hand, did not match the experimental values significantly.

The reason for the above results can be attributed to the following:

(1) Rock unloading strength may be affected by a variety of factors and there may be complex interactions between these factors. The RF model can naturally handle multi-categorization or multi-labeling tasks such as rock strength prediction without the need for one-to-one or one-to-all approaches as in SVM. Therefore, the RF model is more suitable than the SVM model for rock unloading strength predictions.
(2) In addition, the XGBoost model is a highly flexible model that can fit nonlinear relationships well, whereas the RF model and SVM model may not perform well in dealing with certain types of nonlinear relationships; thus, the XGBoost model outperforms the RF model and SVM model in terms of prediction accuracy.
(3) Finally, both the PSO-XGBoost hybrid model and GS-XGBoost hybrid model improved the prediction accuracy and generalization ability by optimizing the hyperparameters of the XGBoost model. In particular, the PSO-XGBoost model achieved a fast and extensive search through the particle swarm optimization algorithm, and it is able to find a near-optimal combination of parameters in a shorter time. In contrast, the GS-XGBoost model systematically explores the predefined parameter space through the grid search method, which is able to find the global optimal solution but is less efficient when the parameter space is large. In summary, with respect to the prediction performance, PSO-XGBoost hybrid model > GS-XGBoost hybrid model > XGBoost model.

### 4.4. Feature Importance Analysis

Feature importance represents the degree of contribution of each feature to the prediction result of the model. The higher the importance of a feature, the greater the impact of the feature on the prediction results. The process of calculating the feature importance of the XGBoost model is as follows:

(1) Node splitting: During the construction of the tree, the features and splitting points that cause the objective function (e.g., the loss function) to decrease the most are selected at each moment a node splits. This process is achieved by maximizing the gain (Gain).
(2) Gain calculation: The gain indicates the degree of improvement of the objective function before and after the split. Specifically, the gain is the sum of the objective function of the two child nodes after the split minus the objective function of the parent node before the split. The greater the gain, the greater the contribution of the feature in the splitting of this node.
(3) Gain accumulation: Traverse all trees and nodes and accumulate the gain of each feature over all nodes to obtain the total value of each feature.
(4) Normalization: The total gain of each feature is divided by the total gain of all features to obtain the relative importance of each feature.

**Figure 6:**
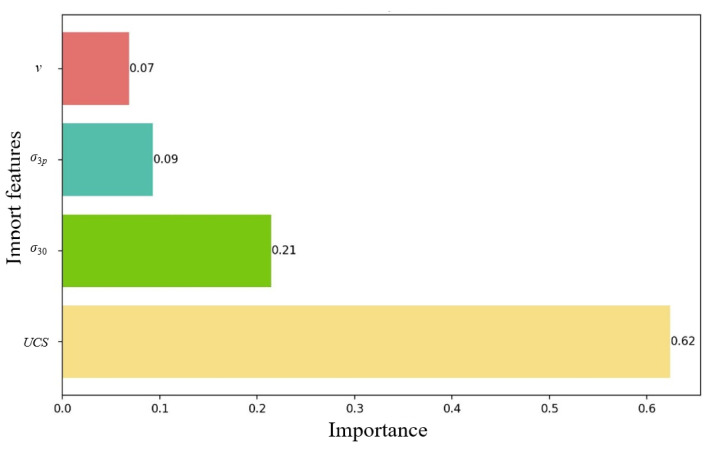
Results of the feature importance analysis.

In practical application, the dataset shown in Table 1 was used, where the input features were *UCS*, *σ*_30_, *σ*_3*p*_, and *v*. The PSO-XGBoost model was applied for regression prediction, and the influence of each input feature on the prediction results was evaluated by feature importance analysis. The specific visualization in Figure 6 shows the percentage of importance of each feature, and the relative contribution of each feature is demonstrated by bars of different colors. The labeling of the values retaining two decimal places makes the graphs clearer and easier to understand. Evidently, for rocks under unloading conditions, the nature of the rock itself, i.e., *UCS*, is the most dominant factor affecting the model generalization performance among the factors considered in this study. This is followed by *σ*_30_, while *σ*_3*p*_ and *v* have the least impact on the model accuracy. The final order of relative importance of features is *UCS* > *σ*_30_ > *σ*_3*p*_ > *v*. Feature importance analysis helps one to understand the decision-making mechanism of the model, such that the model can be improved according to the actual situation. For example, it can be considered for eliminating low importance features to simplify the model or to perform further analysis and optimization of the data for features with high importance. However, it cannot directly infer the global impact of features on the entire dataset, and other explanatory methods can be considered in future research.

### 4.5. Discussion

Theoretically, the PSO-XGBoost hybrid model is capable of globally searching to find the optimal parameter combination of the XGBoost model, which contributes to the prediction accuracy of the model. The comparison between the predictions and the measurements also numerically demonstrates that the performance of the PSO-XGBoost hybrid model is optimal in all the evaluation indexes among the above models, which implies that the prediction accuracy of the rock unloading strength based on the PSO-XGBoost hybrid model is greatly improved. However, the PSO-XGBoost hybrid model has some limitations in spite of its advantages in rock unloading strength prediction. First, the PSO algorithm is an iterative process that requires a large number of computational resources for training multiple models to find optimal parameters. For large-scale datasets or complex models, this can be very time consuming, and there is also a risk of falling into local optimal solutions. Next, the number of samples used for model training in this study was insufficient. Although the PSO-XGBoost hybrid model has satisfactory adaptability to smaller-scale datasets, the prediction results still heavily rely on high-quality input data, and changes in the type and nature of rocks may affect the generalization ability. Therefore, these two aspects should be emphasized in subsequent studies. For example, it is feasible to incorporate more optimized models, such as deep learning models or other machine learning models, as well as using AutoML tools to automate the entire model selection, tuning, and validation process based on the quality of the dataset, thus further improving the model accuracy. Meanwhile, the collection of more rock samples, as well as detailed rock characterization data, can also enrich the dataset and avoid the effect of rock specificity.

## 5. Conclusions

(1) The complexity of the rock unloading environment makes it difficult to perform accurate strength predictions. In this study, the particle swarm optimization (PSO) algorithm was combined with the extreme gradient boosting (XGBoost) algorithm to propose a new rock unloading strength prediction model. Through using PSO to automate the parameter optimization search, the dependence on experience can be reduced and the prediction accuracy and efficiency of the XGBoost model can be improved.
(2) Five indicators (R^2^, MAE, MAPE, MSE, and RMSE) were selected to evaluate the generalization performance of the proposed PSO-XGBoost hybrid model against the other four mainstream models (GS-XGBoost, XGBoost, RF, and SVM), in which the PSO-XGBoost hybrid model outperformed the other models in all the evaluation indicators. The generalization performance was ranked as PSO-XGBoost hybrid model > GS-XGBoost hybrid model > XGBoost model > RF model > SVM model.
(3) For the four input features of the uniaxial compressive strength *UCS*, initial confining stress *σ*_30_, failure confining stress *σ*_3*p*_, and unloading velocity *v*, the gain of each feature was calculated for the importance analysis to assess the importance of each feature on the performance of the PSO-XGBoost hybrid model. The importance order of these features for the unloading strength of rock was obtained as follows: *UCS* > *σ*_30_ > *σ*_3*p*_ > *v*.

## Figures and Tables

**Table 1:** Dataset of rock unloading tests.

Rock Type	Characteristics	Properties	*UCS*/MPa	*σ*_30_/MPa	*v*/MPa/s	*σ*_3*p*_/MPa	*σ*_1*p*_/MPa
Feldspar sandstone [29]	Medium-fine grained feldspathic sandstone, greenish gray, and pore-based cementation.	-	56.94	15	0.033	10.15	81.15
			56.94	25	0.033	14.8	108.97
			56.94	35	0.033	20.7	122.35
			56.94	45	0.033	23.5	139.27
			56.94	15	0.033	10.8	88.2
			56.94	25	0.033	16.8	113.69
			56.94	35	0.033	21.75	129.46
			56.94	45	0.033	28.1	144.85
			56.94	15	0.033	11.6	94.51
			56.94	25	0.033	18.7	122.16
			56.94	35	0.033	25.6	134.38
			56.94	45	0.033	32.2	149.8
Coal [30]	Collected from the No. 16 coal seam in the Yangcun coal mine.	Wave velocities: 1.9~2.0 km/s	20.3	4	0.02	2.28	33.1
			20.3	7	0.02	4.35	40.5
			20.3	10	0.02	6.07	56.61
			20.3	4	0.05	0.62	29.77
			20.3	7	0.05	3.8	38.79
			20.3	10	0.05	4.53	53.28
			20.3	4	0.08	0.6	26.8
			20.3	7	0.08	1.61	36.81
			20.3	10	0.08	4.53	47.68
			20.3	10	0.11	1.55	45.65
			20.3	10	0.14	1.39	43.63
Traditional coal [31]	Made by coal particles with a small size	Cohesion: 1.72 MPa; Friction: 35.06°	1.66	2	0.005	1.69	10.58
			1.66	4	0.005	3.02	17.04
			1.66	6	0.005	4.4	22.92
			1.66	8	0.005	5.75	27.56
Newly reconstituted coal [31]	Made by small coal blocks and coal powder	Cohesion: 1.18 MPa; Friction: 35.06°	0.95	2	0.005	1.61	9.54
			0.95	4	0.005	3.66	15.01
			0.95	6	0.005	4.74	21.62
			0.95	8	0.005	6.31	26.13
Mudstone [32]	Collected from a tunnel project in a Lower Jurassic interlayered mudstone–sandstone stratum.	Density: 2.63 g/cm^3^; Water content: 2.14%; Porosity: 1.67%; Wave velocities: 2.663 km/s;	49.01	10	0.05	4.8	49.81
			49.01	10	0.05	5.25	48.27
			49.01	20	0.05	14.82	73.48
			49.01	20	0.05	14.8	75.35
			49.01	25	0.05	17.49	99.12
			49.01	25	0.05	17.27	101.76
Sandstone [32]		Density: 2.68 g/cm^3^; Water content: 2.53%; Porosity: 6.03%; Wave velocities: 4.214 km/s.	97.88	10	0.05	6.65	106.55
			97.88	10	0.05	6.89	123.78
			97.88	20	0.05	20	144
			97.88	20	0.05	14.98	139.29
			97.88	25	0.05	19.06	147.91
			97.88	25	0.05	19.26	147.01
Shale [33]	Deposited in the period as the gas-bearing shale of the deep Longmaxi Formation.	Elastic modulus: 19.5 GPa; Poisson Ratio: 0.19; Tensile strength: 10.23 MPa.	124.26	60	0.4	51.71	326.37
			124.26	60	0.6	53.7	333.35
			124.26	60	0.8	54.74	343.34
			124.26	60	1.0	54.15	355.51
Marble [34]	Gray-white marbles belonging to the Baishan group (T2b) of the Triassic strata.	Density: 2.72 g/cm^3^; Wave velocities: 5.338~6.354 km/s.	79.15	10	0.01	2.2	95.26
			79.15	10	0.05	0	74.27
			79.15	20	0.01	10.15	118.71
			79.15	20	0.05	0	105.74
			79.15	40	0.01	22	177.07
			79.15	40	0.05	11.2	127.61
Shale (0°) [27]	The Paleozoic lower Silurian Longmaxi Formation of the middle and upper Yangtze regions.	Elastic modulus: 25.1 GPa; Poisson Ratio: 0.111 Cohesion: 28.88 MPa; Friction: 52.3°	146.41	20	0.008	19.64	263.7
			146.41	20	0.033	19.57	261.5
			146.41	20	0.067	15.53	255.3
			146.41	20	0.1	14.26	250.7
Shale (60°) [27]		Elastic modulus: 23.8 GPa; Poisson Ratio: 0.113; Cohesion: 23.96 MPa; Friction: 41.7°;	100.52	20	0.008	17.37	237.2
			100.52	20	0.03	13.69	218.5
			100.52	20	0.06	9.34	197.5
			100.52	20	0.1	16.53	174.2
Shale (90°) [27]		Elastic modulus: 37.4 GPa; Poisson Ratio: 0.136; Cohesion: 53.35 MPa; Friction: 45.6°.	229.33	20	0.008	18.03	336.2
			229.33	20	0.03	13.56	351.5
			229.33	20	0.06	14.18	316.4
			229.33	20	0.1	7.25	322.7
Granite [35]	Fine-to-medium-grained granite with a massive structure.	Density: 2.64 g/cm^3^; Wave velocities: 3.6~4.4 km/s.	138	10	0.02	2.3	190.6
			138	20	0.02	10.2	266.9
			138	40	0.02	24.4	366.8
			138	60	0.02	56.8	404.3
Coal [36]	Taken from No. 3 coal and its roof sandstone in Yangcun Coal Mine, Jining, Shandong, China.	-	20.91	4	0.02	2.3	34.04
			20.91	4	0.05	0.63	30.65
			20.91	4	0.08	0.61	27.6
			20.91	7	0.02	4.39	41.62
			20.91	7	0.05	3.84	39.88
			20.91	7	0.08	1.63	37.89
			20.91	10	0.02	6.13	58.19
			20.91	10	0.05	4.58	54.79
			20.91	10	0.08	4.58	49.02
			20.91	10	0.11	1.57	46.99
			20.91	10	0.14	1.4	44.91
Sandstone [36]		-	131.34	4	0.02	1.24	136.51
			131.34	4	0.05	0.01	132.96
			131.34	4	0.08	0.01	124.02
			131.34	7	0.02	4.36	143.25
			131.34	7	0.05	1.88	138.24
			131.34	7	0.08	0	125.75
			131.34	10	0.02	7.25	158.85
			131.34	10	0.05	4.99	146.04
			131.34	10	0.08	2.75	141.38
			131.34	10	0.11	2.7	135.47
			131.34	10	0.14	0	132.21
			131.34	13	0.05	6.82	156.75
			131.34	16	0.05	8.69	168.11
			131.34	19	0.05	11.56	192.53
Gas-bearing coal-sandstone [37]	Taken from the No. 15 coal seam and its roof rock of the Xinjing coal mine in Shanxi Province, China.	-	10.88	4	0.01	1.81	25.19
			10.88	4	0.01	2.11	16.87
			10.88	4	0.01	2.72	15.01
			10.88	7	0.01	2.41	18.17
			10.88	10	0.01	3.23	23.98
Granite [38]	Dense granite sample, mainly composed of quartz, feldspar, hornblende, and black mica.	-	228.7	5	0.01	0	180.2
			228.7	10	0.01	0	212.6
			228.7	15	0.01	3	265.6
			228.7	20	0.01	4.75	301.5
Dry sandstone [39]	Obtained from a quarry in Yunnan Province	Density: 2.388 g/cm^3^; Wave velocities: 3.305 km/s.	117.35	10	0.05	1.52	118.54
			117.35	20	0.05	14.82	151.15
			117.35	30	0.05	15.51	181.04
			117.35	40	0.05	20.01	200.74
Saturated sandstone [39]		Density: 2.457 g/cm^3^; Wave velocities: 3.373 km/s.	94.76	10	0.05	4.56	91.7
			94.76	20	0.05	10.56	120.91
			94.76	30	0.05	19.73	144.01
			94.76	40	0.05	21.61	153.19
Intact sandy slate [40]	Jointed sandy slate of the underground plant of the Kara hydroelectric power station.	Density: 2.68~2.75 g/cm^3^; Wave velocities: 2.9~3.4 km/s.	81.15	5	0.1	4.09	119.75
			81.15	10	0.1	7.03	147.15
			81.15	15	0.1	9.06	181.02
			81.15	20	0.1	9.06	222.10
Fractured sandy slate [40]		Cohesion: 0.114 MPa; Friction: 27.605°	57.74	5	0.1	1.69	95.63
			57.74	10	0.1	9.04	120.49
			57.74	15	0.1	13.15	146.09
			57.74	20	0.1	18.43	195.15
Sandstone [41]	Taken from the tunnel construction site and consisted of quartz, feldspar, and mica.	Density: 2.57 g/cm^3^; Porosity: 0.68%; Particle size: 0.018~0.540 mm.	149.44	20	0.1	15.96	183.54
			149.44	30	0.1	24.88	224.84
			149.44	40	0.1	33.49	255.71
			149.44	50	0.1	40.35	273.82
Quartz mica schist (vertical) [42]	Gray-black in appearance with glittering minerals that were thinly layered and had significant lamellar structures.	Cohesion: 12.40 MPa; Friction: 33.76°;	55.41	30	0.05	16.99	106.07
			55.41	40	0.05	18	127.31
			55.41	50	0.05	37.27	183.06
			55.41	60	0.05	38.09	202.64
Quartz mica schist (parallel) [42]		Cohesion: 14.39 MPa; Friction: 35.11°.	46.42	30	0.05	16.66	98.19
			46.42	40	0.05	25.02	134.02
			46.42	50	0.05	35.89	164.83
			46.42	60	0.05	45.87	191.06
Granite [43]	Grayish white	Density: 2.5 g/cm^3^; Wave velocities: 3.2~3.8 km/s.	80	10	0.05	5.4	192.4
			80	20	0.05	12.1	250
			80	30	0.05	18.6	304.3

**Table 2:** Evaluation indicators of the predictive models.

Evaluation Indicator	Computational Formula	Evaluation Criteria
Coefficient of determination	$R^{2}=1-\frac{\sum_{j=1}^{n}{\left(\sigma_{test}-\sigma_{pre}\right)}^{2}}{\sum_{j=1}^{n}{\left(\sigma_{test}-\sigma_{pre}^{mean}\right)}^{2}}$	Larger R^2^, better performance
Mean square error	$\mathrm{MSE}=\frac{1}{n}\sum_{j=1}^{n}{\left(\sigma_{test}-\sigma_{pre}\right)}^{2}$	Smaller MSE, better performance
Root mean square error	$\mathrm{RMSE}=\sqrt{\frac{1}{n}\sum_{j=1}^{n}{\left(\sigma_{test}-\sigma_{pre}\right)}^{2}}$	Smaller RMSE, better performance
Mean absolute error	$\mathrm{MAE}=\frac{1}{n}\sum_{j=1}^{n}\left|\sigma_{test}-\sigma_{pre}\right|$	Smaller MAE, better performance
Mean absolute percentage error	$\mathrm{MAPE}=\frac{1}{n}\sum_{j=1}^{n}\left|\frac{\sigma_{test}-\sigma_{pre}}{\sigma_{test}}\right|\times 100\%$	Smaller MAPE, better performance

**Table 3:** Calculation of the evaluation indicators for different model prediction results.

Evaluation Indicators	Prediction Performance				
	RF	SVM	XGBoost	GS-XGBoost	PSO-XGBoost
R^2^	0.89	0.22	0.92	0.93	0.98
MAE	18.66	68.63	16.74	14.95	8.80
MAPE	13.02	125.33	11.60	10.40	6.51
MSE	1158.51	8163.92	858.29	702.65	198.10
RMSE	34.04	90.35	29.30	26.51	14.07

## Data Availability

The data used to support the findings of this study are available from the corresponding authors upon request.
